# Protective Effects of *Momordica charantia* Fruit Extract on Male Sexual Dysfunction and Testicular Damage in Rats Induced by Chronic Unpredictable Stressors

**DOI:** 10.3390/life15101559

**Published:** 2025-10-04

**Authors:** Therachon Kamollerd, Suwit Uopasai, Tarinee Sawatpanich, Nongnut Uabundit, Supatcharee Arun, Nareelak Tangsrisakda, Chayakorn Taoto, Chadaporn Chaimontri, Natthapol Lapyuneyong, Wipawee Thukhammee, Sararat Innoi, Sitthichai Iamsaard

**Affiliations:** 1 Department of Anatomy, Faculty of Medicine, Khon Kaen University, Khon Kaen 40002, Thailand; therachon_k@kkumail.com (T.K.); tarinee@kku.ac.th (T.S.); nongua@kku.ac.th (N.U.); supatar@kku.ac.th (S.A.); nareelak@kku.ac.th (N.T.); chayakorntaoto@kkumail.com (C.T.); chadaporn_chaimontri@kkumail.com (C.C.); l.natthapol@kkumail.com (N.L.); sararat.i@kkumail.com (S.I.); 2 Department of Anatomy, Faculty of Veterinary Medicine, Khon Kaen University, Khon Kaen 40002, Thailand; suwuop@kku.ac.th; 3 Department of Physiology, Faculty of Medicine, Khon Kaen University, Khon Kaen 40002, Thailand; wipath@kku.ac.th

**Keywords:** *Momordica charantia*, chronic stress, sexual behavior, testis, apoptosis

## Abstract

Chronic stress (CS) is known to induce testicular oxidative stress and apoptosis. Fruit of *Momordica charantia* (FMC) has antioxidant capacity to protect tissue damage, but its effect on sexual activity and testicular damage caused by CS has never been documented. This study aimed to investigate the impact of FMC extract against testicular damage and sexual dysfunction in chronic unpredictable stress (CUS) rats. Rats were divided into four groups and pretreated with vehicle or FMC (40 and 80 mg/kg) before CUS induction for 56 days. Sexual behaviors, serum hormones, and sperm quality were analyzed. Testes were collected to determine daily sperm production (DSP), malondialdehyde (MDA) level, and expressions of cleaved apoptotic proteins. Testicular DNA fragmentation, as revealed by TUNEL and morphometric analysis, was observed. FMC improved sexual activity, increased testosterone levels, and sperm count with improvements in DSP, testicular morphometrics, and MDA levels. Moreover, TUNEL-positive cells and expression of caspase 3 in the testis were decreased in FMC rats. FMC has antioxidant potential and could protect against male sexual dysfunction and testicular damage caused by stress-related apoptosis. It is a potential extract to be developed as a supplement in preventing CS-male subfertility. However, future studies about the optimal dose and clinical trials are required.

## 1. Introduction

Chronic stress (CS) is widely known to disrupt homeostasis, leading to various pathologies in the nervous, endocrine, cardiovascular, digestive, urinary, and reproductive systems [1,2,3,4]. Particularly, many studies demonstrated the adverse effect of CS on the male reproductive system, including atrophic seminiferous tubules, testicular apoptosis, low sperm quality, reduced daily sperm production, decreased testosterone levels, low sexual performances (mounting, intromission, and ejaculation), leading to male infertility or subfertility [5,6,7,8,9,10]. In addition, the altered expressions of key proteins involving testicular function, including tyrosine phosphorylated proteins, steroidogenic acute regulatory (StAR) protein, CYP11A1, androgen receptor (AR), and heat shock protein 70 (HSP70) in CS testes have been reported [6,11,12]. Moreover, CS significantly elevated markers of oxidative stress and apoptosis, such as lipid peroxidation or malondialdehyde (MDA) levels, as well as caspase 3 and caspase 9 [8,13,14,15].

Since some recent drugs used for CS treatments have been reported for their side effects on other systems, including the male reproductive system, many researchers have attempted to investigate the protective effects of alternative treatments, especially herbal medicines, for improving structural and functional damage in the testis. *Momordica charantia* (MC), commonly known as Bitter gourd, is a tropical plant commonly found in Southeast Asia, including Thailand. Many parts of the MC tree have been used in traditional medicine to treat many diseases, especially the fruit of the MC (FMC). Previously, FMC has been shown to have antioxidant capacity and contain many bioactive compounds such as momordicin I, saponin, flavonoids, and phenolic compounds [16,17,18]. In addition, FMC has many pharmacological properties, including hypoglycemic, antibacterial, antiviral, anthelmintic, anti-inflammatory, wound-healing, and antioxidant effects [19,20]. In animal models, FMC has been demonstrated to have protective effects against some diseases and physiological pathologies such as diabetes, cardiac fibrosis, and testicular [16,21,22]. Moreover, a prior study showed that FMC extract could protect against changes in tyrosine phosphorylation in the epididymal tissue and fluid of chronic stress rats [23]. That study implies that FMC may facilitate sperm physiological maturation. From previous literature, the diminished sexual behavior can negatively impact fertility [6,10]. While previous studies suggest that the FMC may protect against testicular and epididymal damage [22,23], no study has evaluated its therapeutic effects against the impact of CS on both sexual behavior and testicular apoptosis. Therefore, this study aimed to investigate the preventive effects of FMC on the decline of sexual performance and testicular damage by assessing its role on apoptotic protein expression and oxidative stress markers in rats induced with chronic unpredictable stress (CUS).

## 2. Materials and Methods

### 2.1. Animals and Ethics

Thirty-two adult male (10 weeks old) and twenty female (10 weeks old) Wistar rats were purchased from Nomura Siam International, Bangkok, Thailand. The animals were housed in the 7.5 × 48 × 21 cm plastic cages (4 rats per cage) under the temperature of 23 ± 2 °C, light/dark cycle at 12 h, light intensity at 350–400 lux, relative humidity at 30–60%, and sound level of <85 dB. All rats were fed commercial pellet food and had access to water ad libitum. After acclimatization for 7 days, male rats were trained for copulatory behavior capacities (mounting, intromission, and ejaculation) with hormonally induced estrous females. The training session was performed as described in the previous study [24]. Then, they were randomized and divided into 4 groups (8 in each group), including control, chronic unpredictable stress (CUS), FMC40 + CUS, and FMC80 + CUS, respectively. In the control and CUS groups, rats received only vehicle via oral gavage. The co-administered groups were daily treated with FMC dissolved in DW at doses of 40 or 80 mg/kg/day for 56 days based on the period of a spermatogenesis cycle and sperm transit into the epididymis [25]. After 30 min of FMC administration via the oral gavage needle, all animals in the stress groups were induced for modified CUS. Briefly, the animals were performed each day by a single stressor that was randomized from different 9 stressors: (1) food deprivation for 24 h, (2) water deprivation for 24 h, (3) cold water swimming for 5 min, (4) change in cage mate for 12 h, (5) tail pinch was applied 1 cm proximal to the tip of the tail for a duration of 1 min, (6) 45° cage tilling for 12 h, (7) overcrowding of cage for 12 h, (8) wet bedding for 12 h, and (9) restraint for 4 h. Each stressor was randomly selected and performed for 3–4 rounds within 56 days of CUS induction. At the end of the experiment, the male rats were tested for male sexual behaviors before sacrifice to collect blood and other reproductive organs for further analyses. The Institutional Animal Care and Use Committee of Khon Kaen University has approved the animal ethics of this study (code: IACUC-KKU-63/65).

### 2.2. Antioxidant Capacity Assays

#### 2.2.1. Total Phenolic Content Estimation

Total phenolic content (TPC) of FMC was evaluated by the modified Folin–Ciocalteu colorimetric method [26]. Briefly, the extract was diluted in distilled water (DW) and mixed with Folin reagent (Sigma-Aldrich, Burlington, MA, USA) and added with 7% sodium carbonate before incubation for 90 min to develop a color reaction. Then, the mixture solution was measured for the color absorbance at 760 nanometers using the spectrophotometer. The TPC was expressed as milligrams of gallic acid equivalent (GAE) per gram of sample (mg GAE/g sample).

#### 2.2.2. Flavonoid Content Determination

Flavonoid content (FC) in FMC was determined based on a previous study [26]. In brief, FMC was dissolved in DW and added with sodium nitrite before mixing with 10% aluminum chloride. After 6 min of incubation, 1 M sodium hydroxide was added. Then, the color absorbance at 510 nanometers was measured by the spectrophotometer. The FC was expressed as milligram catechin per gram sample (mg catechin/g sample).

#### 2.2.3. The 2,2-Diphenyl-1-picrylhydrazyl (DPPH) Radical Scavenging Capacity Assay

This study determined the radical scavenging capacity of FMC using the following assays.

##### Trolox Equivalent

The free radical scavenging effect of FMC was compared to that of Trolox using the DPPH assay [27]. Briefly, the 0.1 mM DPPH in 95% ethanol was prepared, mixed with FMC or other antioxidant control agents (ascorbic acid, α-tocopherol, and BHT). Then, the solutions were incubated for 30 min. After that, their absorbance was read at 517 nanometers by the spectroscope. The free radical scavenging effect was estimated by percentage of DPPH radical scavenging activity as the following formula: DPPH radical scavenging activity (%) = [(absorbance of the negative control—absorbance of FMC or other control agents)/absorbance of the negative control] × 100. The standard curve was plotted from Trolox concentrations from 2.0 to 6.0 μg/mL.

##### Inhibitory Concentration at 50% (IC50)

The free radical scavenging effect of FMC at IC50 was measured as described in a previous study [27]. The FMC and antioxidant control agents (ascorbic acid, α-tocopherol, and BHT) were prepared for 5 ascending concentrations. Then, they were added with 0.1 M DPPH before incubation for 30 min. The absorbance at 517 nanometers was determined and used for the calculation of % DPPH radical scavenging activity. Subsequently, % DPPH radical scavenging activity was plotted and calculated for IC50, which was reported as milligram per milliliter (IC50 mg/mL).

#### 2.2.4. Ferric Reducing Antioxidant Power (FRAP) Assay

The antioxidant activity of FMC was measured with the FRAP assay [28]. Briefly, 300 mM acetate buffer was mixed with 20 μM FeCl_3_·6H_2_O and 40 μM TPTZ (2,4,6-tris(2-pyridyl)-s-triazine) in HCl to prepare the FRAP reagent. Then, the FMC or other antioxidant agents (ascorbic acid, α-tocopherol, and BHT) were mixed with the FRAP reagent before incubation at 37 °C for 4 min. The absorbance of the color mixture was read at a wavelength of 595 nanometers. The antioxidant activity was compared to a standard curve of Ferrous sulfate (FeSO_4_·7H_2_O) from 0.1 to 1 mmol. Its activity was reported as micromole of ferrous ion per 1 g of sample (μmol of Fe (II)/1 g of sample).

### 2.3. Nuclear Magnetic Resonance (NMR) Analysis

The metabolic profiles of FMC were analyzed using nuclear magnetic resonance (NMR) at the Khon Kaen University International Phenome Laboratory (KKUIPL), Thailand. In brief, the 500 mg FMC was mixed with 1000 µL of NMR buffer containing 0.08% trisodium phosphate (TSP), 0.075 mM Na_2_HPO_4_, and 2 mM NaN_3_ diluted in D_2_O (pH 7.4). Then, the mixture was sonicated for 10 min (3 times) and centrifuged at 13,000 rpm at 4 °C for 10 min (2 cycles) to separate the particles from the supernatant. Then, the supernatant was filtered through the microfilter. The supernatant (560 µL) was transferred into an NMR tube. Subsequently, the ^1^H NMR spectra were acquired using a 600 MHz NMR spectrometer (Bruker, Billerica, MA, USA). The Matrix Laboratory (MATLAB) software was used for NMR spectra adjustment, and the trisodium phosphate (TSP) peak was set at 0 ppm. Then, the water peak (4.5 to 5.0 ppm) and TSP peak (−1 to 0.551 ppm) regions were systematically removed. The metabolite identification was performed using Statistical Total Correlation Spectroscopy (STOCSY) in MATLAB R2015a software (MathWorks, Natick, MA, USA) and further confirmed with an in-house database, ChenomxNMR Suite version 9.0 (Chenomx, Edmonton, AB, Canada), and Human Metabolome Database (HMDB).

### 2.4. Sexual Behavior Test

The sexual behavior test in rats was conducted as described previously [24]. Briefly, the estrus phase was induced in adult female rats using 20 μg estradiol benzoate hormone (Sigma-Aldrich, Burlington, MA, USA) for 52 h and 1000 μg progesterone hormone (Sigma Aldrich, USA) for four hours before the test. The estrous status was confirmed by presenting cornified epithelial cells in the vaginal lavage observed from vaginal smear [29]. The test was performed in the dark room at 7.00–11.00 p.m. on day 56. To familiarize the animals with the testing area, a male rat from each group was placed in the arena (40 × 60 × 40 cm, plastic box with Plexiglass on the front side) for 10 min. Then, the receptive female rat was placed into the same arena. After coupling, sexual behaviors were recorded for 30 min by using the security cameras (lateral view) and the world wide web camera (ventral view) operated via the video recording program (OBS studio program, Free Software Foundation, Inc., Boston, MA, USA). The determination of sexual performances in this study was explained by previous works [24,30]. Those sexual behavior parameters included the mount frequency (MF), mount latency (ML), intromission frequency (IF), and intromission latency (IL), respectively.

### 2.5. Sample Collections

At the end of the experiment, all rats were anesthetized with thiopental sodium injection at 40 mg/kg before euthanasia by cervical dislocation. For hormone analysis, the blood was punctured via the left ventricle and centrifuged at 13,000 rpm, 10 min at 4 °C to separate the serum from blood cells. Then, the epididymis, vas deferens, and testes were collected and weighed. The right testis was fixed in 10% formalin, while the left testis (kept at −20 °C) was evaluated for protein expression. To collect sperm, the sperm masses from the cauda epididymis and vas deferens were squeezed and resuspended in 1 mL of PBS (37 °C) before evaluating their qualities as previously described in previous work [30].

### 2.6. Serum Hormone Measurement

The blood serum of male rats was analyzed for cortisol and testosterone levels using an electrochemiluminescence immunoassay (Roche Cobas^®^ e801 Immunoassay, Basel, Switzerland) at the Specimen Center, Srinagarind Hospital, Khon Kaen University, Thailand. In brief, the serum was incubated with cortisol- or testosterone-specific biotinylated monoclonal antibody before binding with streptavidin-coated microparticles. Then, the mixtures were subjected to the measuring cells and induced with an electrode. The chemiluminescent emission was measured by a photomultiplier and calculated for hormonal concentrations (expressed as ng/dL).

### 2.7. Sperm Quality Assays

#### 2.7.1. Sperm Viability

The suspended sperm (50 μL) was mixed with eosin–nigrosin solution (Baso diagnostic, Zhuhai, China) in a 1:1 ratio before incubation for 30 s at 37 °C. Then, the 20 μL of the sperm mixture was smeared on the glass slide before observation and counting under a light microscope (400× magnification). A total of 400 sperm were observed and counted for their live (unstained) and dead (pink-stained) statuses.

#### 2.7.2. Sperm Concentration

The 20 μL of fixed sperm was loaded onto hemocytometer counting chambers and counted under a light microscope at 400× magnification. Then, the sperm concentrations (million sperm cells/mL) were calculated from the formulation as described in a previous study [30].

### 2.8. Daily Sperm Production (DSP)

The determination of DSP was modified from previous reports [30,31]. Each frozen testis was decapsulated and bisected before being mixed with the extraction buffer (0.9% NaCl containing 0.5% TritonX100). Then, the testicular parenchyma, including seminiferous tubules, was minced and incubated for 10 min on ice. After that, the testicular lysate was further sonicated at 130 watts for 30 times (1 s per time) by using the ultrasonic probe homogenizer (Cole-Parmer Instrument Company, Vernon Hills, IL, USA). Subsequently, the homogenized testicular lysate was mixed with eosin Y aqueous solution (Bio-Optica Milano s.p.a, Milano, LOM, Italy) and incubated for 5 min before loading (20 µL) on the hemocytometer counting chamber. The sperm head at steps 17–19 of spermiogenesis were counted under a light microscope at 100× magnification in triplicate, as also shown in Figure 3A. The DSP was calculated from the following: the total concentration of spermatid heads is divided by the weight of decapsulated testis before dividing by 6.3 and expressed as DSP/g testis/day [32].

### 2.9. Testicular Protein Preparation and Immuno-Western Blotting

The left testis was decapsulated to collect its parenchyma before mixing with lysis buffer (1× radioimmunoprecipitation assay [RIPA] solution [Cell Signaling Technology, USA] containing protease inhibitor cocktails [Sigma-Aldrich, USA]). Then, it was homogenized, sonicated, and centrifuged at 4 °C and 14,000 rpm for 15 min to collect the supernatant. The total protein in testicular supernatant lysate was measured by using the NANO drop spectrophotometer (ND-100, Nanodrop technologies, Wilmington, DE, USA) at a wavelength of 280 nm.

For Western immunoblotting, 150 μg of total protein from each animal was separated with 10% separating SDS-PAGE gel. Subsequently, the separated proteins were transferred to a nitrocellulose membrane (Bio-Rad Laboratories, Inc., Hercules, CA, USA) at 110 volts for 120 min before blocking of non-specific binding proteins with 5% skim milk (dissolved in 0.1% TBST) at room temperature. To probe apoptotic markers, the blot was incubated with individual primary antibody: anti-Caspase 3 (Cat. No. SC-7272, Santa Cruz, Dallas, TX, USA) or anti-Caspase 9 (Cat. No. SC-56076, Santa Cruz, Dallas, TX, USA) or anti-HSP70 (Cat. No. MAB 3516, Merck, Rahway, NJ, USA) or anti-CYP11A1 (Cat. No. sc-18040, Santa Cruz, Dallas, TX, USA) or anti-StAR (Cat. No. HPA023644, Merck, Rahway, NJ, USA) diluted in 5% skimmed milk diluted in 0.1% TBST (1:3000) at 4 °C for overnight. The anti-β actin was used as an internal control (Cat. No. sc-47778, Santa Cruz, Dallas, TX, USA). Then, unbound antibodies were washed by using 0.1% TBST for 5 min, 3 times before incubating with the specific secondary anti mouse antibody (1:10,000, Cat. No. AP160P, Merck, Rahway, NJ, USA) or anti rabbit antibody (1:10,000, Cat. No. AP132P, Merck, Rahway, NJ, USA) or anti goat antibody (1:10,000, Cat. No. sc-2020, Santa Cruz, Dallas, TX, USA). The specific antigen–antibody complexes on the membrane were incubated with enhanced chemiluminescence (ECL) substrate reagent kit (GE Healthcare Life Sciences, Marlborough, MA, USA) before detecting the positive protein bands under the Gel Documentation 4 (ImageQuant 400, GE Healthcare, Chicago, IL, USA). Then, the intensity of individual proteins was analyzed using the ImageJ program (National Institutes of Health, Bethesda, MD, USA) and calculated as the percentage of protein expression intensity.

### 2.10. Seminiferous Morphometric Analysis

Fixed testes were routinely embedded in a paraffin block, followed by sectioning (7 µm) using an automatic microtome (ERM 3100, Hestion, St. Helens, TAS, Australia). All testicular sections were stained with hematoxylin solution (Cat. No. HX90188374; Merck KgaA, Darmstadt, Germany) and eosin-Y (Cat. No. 05-10002/L; Bio-Optica, Milano, LOM, Italy) before observing under a light microscope (Nikon ECLIPSE E200, Tokyo, Japan). The testicular morphometrics were performed according to a previous study [33]. Briefly, the 4 axes of the seminiferous epithelial heights and tubular diameters at stage IX were measured and analyzed using ImageJ software (version 1.54g, National Institutes of Health, Bethesda, MD, USA).

### 2.11. TUNEL Assay

The terminal deoxynucleotidyl transferase (TdT) dUTP Nick-End Labeling (TUNEL) assay was performed to evaluate apoptosis in the testis. Using TUNEL kits (Cat. No. ab206386, abcam, Cambridge, UK), the testicular sections were permeabilized with proteinase K solution for 20 min and incubated with 3% H_2_O_2_ for 5 min to inactivate the endogenous peroxidases. Then, sections were incubated with TdT equilibration buffer for 30 min and probed with TdT labeling reaction mixture for 1.5 h to catalyze the incorporation of the modified (biotin-labeled) dUTPs (2′-Deoxyuridine, 5′-Triphosphate) to the 3′-OH termini in the apoptotic nuclei. Subsequently, they were incubated in the stopping reaction buffer for 5 min before incubation with the blocking buffer for 10 min. The sections were incubated with 1× conjugates diluted in blocking buffer at room temperature for 30 min to bind dUTPs with streptavidin-horseradish peroxidase. Then, the 3,3′ diaminobenzidine (DAB) solution was applied to the sections for 15 min to locally develop the brownish color in the apoptotic nuclei. The sections were further incubated with methyl green counterstain solution for 3 min before observation under a light microscope.

### 2.12. Statistical Analysis

All data were expressed as mean ± standard deviation (SD). The one-way analysis of variance (ANOVA) and Bonferroni post hoc test were used to compare the differences among groups. The statistical analyses were carried out using GraphPad Prism 10 version 10.4.1 (627) (GraphPad Software, Inc., Boston, MA, USA). A *p*-value less than 0.05 was considered statistically significant.

## 3. Results

### 3.1. Antioxidant Capacity of FMC (Fruit Extract of M. charantia)

The FMC extract was determined to have its antioxidant capacity before using it for further experiments. Total phenolic content (TPC) of FMC was found to be 19.01 ± 0.27 mg GAE/g sample, and its flavonoid content (FC) was approximately 0.31 ± 0.01 mg Catechin/g sample (Table 1). In addition, the DPPH (2,2-diphenyl-1-picrylhydrazyl) radical scavenging activity of FMC was 4.99 ± 0.09, as shown in Table 1. The half maximal inhibitory concentration (IC) of the DPPH radicals from FMC was 2.01 ± 0.01 mg/mL. Moreover, the ferric reducing antioxidant power (FRAP) value of FMC was 23.70 ± 0.82 (µmol of Fe (II)/g). Ascorbic acid, α-Tocopherol, and BHT (Butylated hydroxytoluene) were used as positive control substances (Table 1). The results validated that the FMC extract contains antioxidant activity, which is partly attributed to its phenolic and flavonoid contents.

### 3.2. Metabolite Profiles of FMC by Using ^1^H NMR Spectroscopy

**Figure 1:**
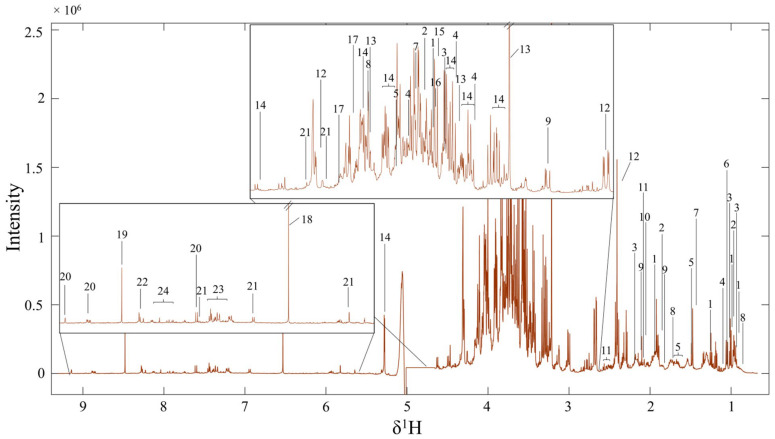
^1^H NMR spectra profiles of FMC extract: (1) isoleucine; (2) 2-aminobutyric acid; (3) valine; (4) propylene glycol; (5) hydroxy-3-methylvaleric acid; (6) methylmalonic acid; (7) alanine; (8) alpha-hydroxy; (9) gamma-aminobutyric acid; (10) acetic acid; (11) saccharopine; (12) malic acid; (13) choline; (14) glucose; (15) propanedinitrile; (16) oxalacetic acid; (17) 6-phosphogluconic acid; (18) fumarate; (19) formic acid; (20) pyrimidine; (21) xanthosine; (22) 4-aminohippuric acid; (23) cinnamic acid; (24) adenine.

The metabolites in FMC extract were analyzed using ^1^H NMR, and the results showed a total of 24 identified metabolites (Figure 1). In addition, the chemical shifts, multiplicity, statistical total correlation spectroscopy (STOCSY), and *p*-value of such metabolites are listed in Table 2. Particularly, the ^1^H NMR spectra in Figure 1 can be divided into 3 regions (X axis), including the aromatic (10-6), sugar (6-3), and organic and amino acid (3-0) regions. As characterized, the metabolite signals found in the aromatic region are fumarate, formic acid, pyrimidine, xanthosine, 4-aminohippuric acid, cinnamic acid, and adenine (Table 2). For the sugar region, choline, glucose, propanedinitrile, oxalacetic acid, and 6-phosphogluconic acid are identified in the FMC. The organic and amino acid regions have the signals of isoleucine, 2-aminobutyric acid, valine, propylene glycol, hydroxy-3-methylvaleric acid, methylmalonic acid, alanine, alpha-hydroxy, gamma-aminobutyric acid, acetic acid, saccharopine, and malic acid, respectively (Figure 1 and Table 2).

### 3.3. FMC Extract Increased Body Weight, Testicular Weight, Sperm Count, and Testosterone Level in Stressed Rats

The final body weight (BW) and its percentage change in the CUS group were significantly decreased compared to the control group (Table 3). The final BW was significantly improved in the FMC80 + CUS group compared to the CUS animals. In addition, the testicular absolute weight was significantly decreased in the CUS group, but it was improved in the FMC-treated groups. In addition, the results showed that the FMC could significantly increase sperm count in CUS rats, but no difference in sperm viability was observed among the treated groups. Although the serum cortisol levels were not different among groups, the testosterone level was significantly decreased in the CUS group and increased in the FMC-treated groups. Testicular MDA levels were significantly lower in the FMC40 + CUS group than in the CUS group, suggesting a decrease in lipid oxidation in FMC FMC-treated group. It was found that both epithelial height and tubular diameter were significantly decreased in the CUS testicular tissue, but they were improved in the FMC-treated groups (Table 3). These findings demonstrated that the FMC extract could alleviate the adverse effects of CUS, leading to improvements in body weight, testicular morphometry, epididymal sperm count, and testosterone levels by decreasing lipid oxidation and oxidative stress.

### 3.4. FMC Extract Increased Male Sexual Behaviors in CUS Rats

**Figure 2:**
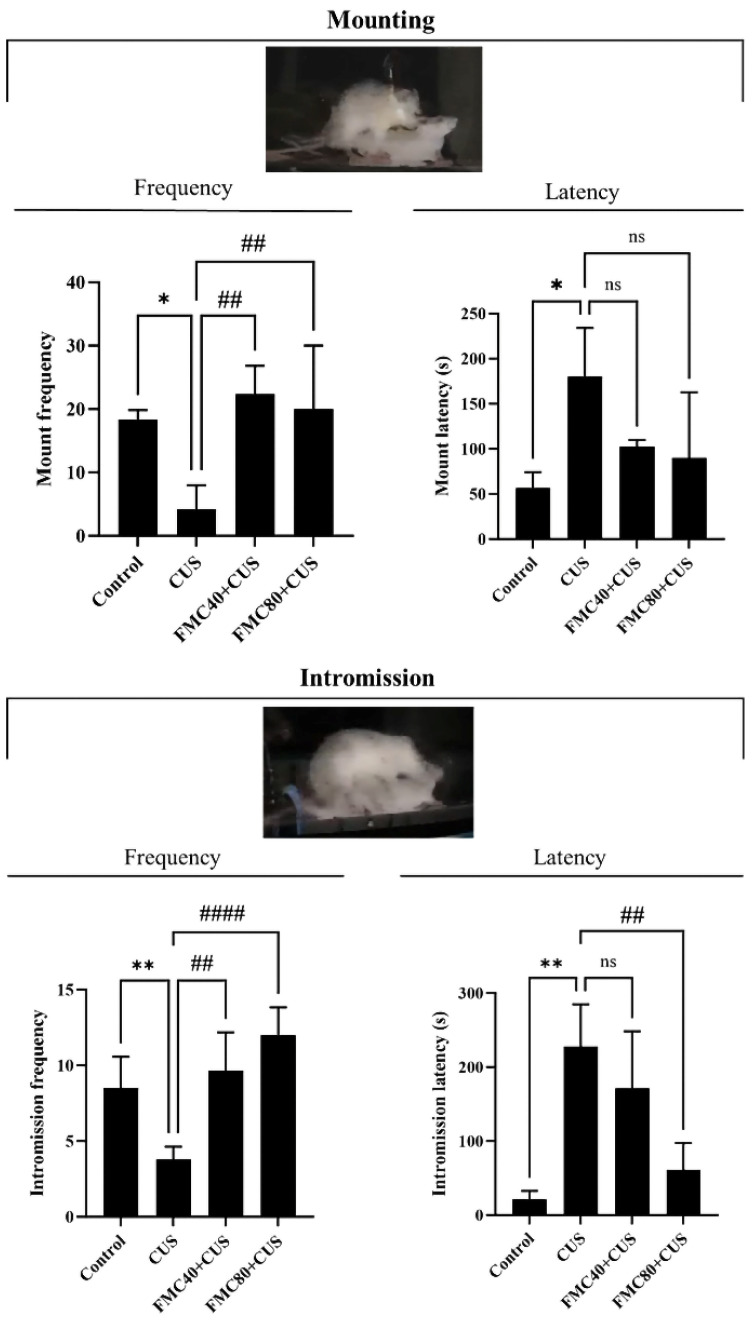
Comparisons of the sexual behaviors (mounting and intromission) of male rats with their frequency and latency. * *p* < 0.05, ** *p* < 0.01 compared to control group, ^##^ *p* < 0.01, ^####^ *p* < 0.0001 compared to CUS, and ns; not significant.

The mount frequency (MF) and intromission frequency (IF) significantly decreased while intromission latency (IL) and mount latency (ML) were elevated in the CUS group compared to the control group (Figure 2). Interestingly, the FMC extracts at doses of 40 and 80 mg/kg could significantly increase the MF and IF. In addition, a dose of 80 mg/kg could significantly decrease the IL when compared to the CUS group (Figure 2). The increasing frequency and decreasing latency of each behavior parameter indicated that the FMC extract could enhance sexual performance in rats under the effects of CUS.

### 3.5. FMC Extract Improves the Daily Sperm Production

**Figure 3:**
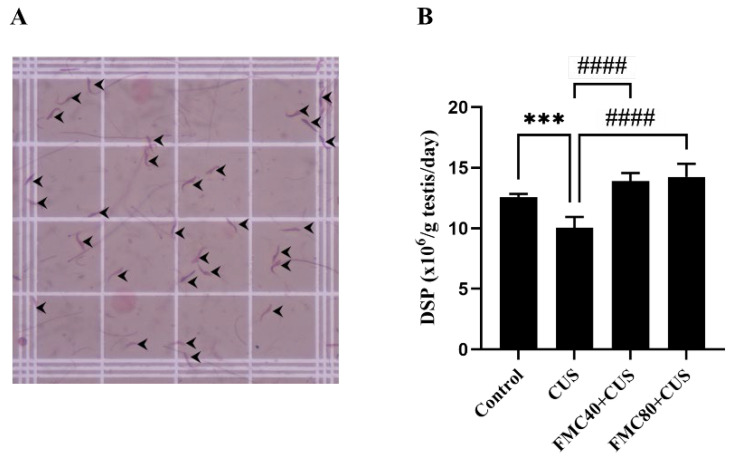
Micrograph showing the testicular sperm head (steps 17–19) stained with eosin dye (black arrow heads) on a hemocytometer counting chamber observed under a light microscope (**A**). Daily sperm production (DSP) (×10^6^/testis/day) of male rats compared among groups (**B**). *** *p* < 0.001, compared to control group, ^####^ *p* < 0.0001 compared to CUS.

It was shown that the DSP in the CUS group was significantly decreased as compared to the control group, whereas the cotreatments with FMC extract (40 and 80 mg/kg) could significantly improve the DSP compared to untreated rats (Figure 3). The improvement of DSP was observed in both FMC-treated groups, indicating a protective effect of FMC on testicular function disruption caused by CUS.

### 3.6. FMC Extract Improved Cleaved Caspase 3 in the CUS Testis

**Figure 4:**
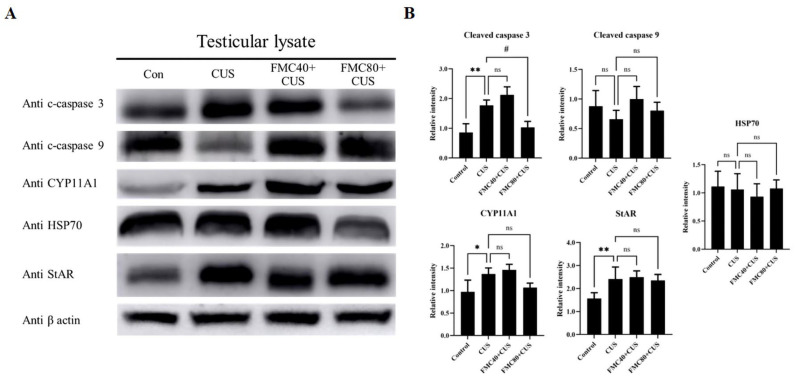
Showing the changes in testicular protein expressions in CUS and/or FMC-CUS treated rats (**A**) and the comparison of the relative intensity of functional testicular proteins among groups (**B**). * *p* < 0.05, ** *p* < 0.01 compared to control group, ^#^ *p* < 0.05 compared to CUS, and ns; not significant. c-caspase 3, cleaved caspase 3; c-caspase 9, cleaved caspase 9; CYP11A1, Cytochrome P450 Family 11 Subfamily A Member 1; StAR, steroidogenic acute regulatory protein; HSP70, heat shock proteins 70.

Figure 4 showed that the expression of cleaved caspase 3 in the CUS group was significantly increased compared to the control and FMC80 + CUS groups, suggesting the anti-apoptotic effect of this FMC dose. However, the expressions of both cleaved caspase 9 and HSP70 were not different among groups. The StAR and CYP11A1 expressions in the CUS rat were significantly increased compared to the control rat (Figure 4). From the expression of these proteins, the FMC extract could ameliorate the testicular apoptosis and maintain testosterone synthesis in the rat testis.

### 3.7. FMC Extract Decreased Apoptosis in the CUS Testicular Tissue

**Figure 5:**
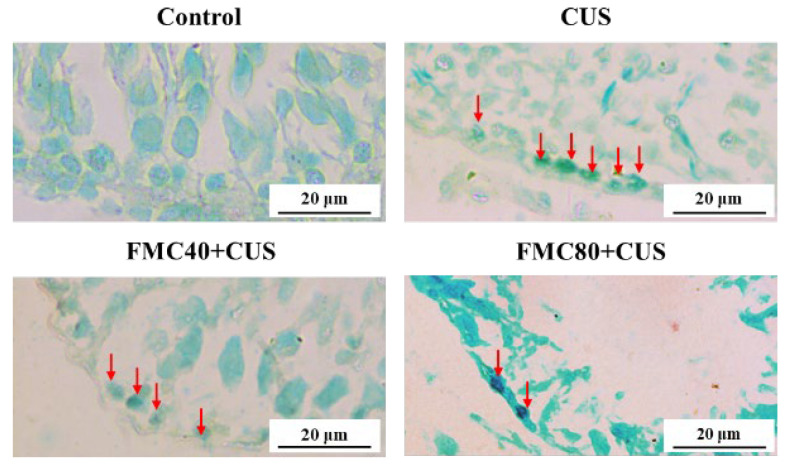
Micrographs showing the testicular tissues with TUNEL-positive cells representing brownish nuclei of the spermatogonial cells (red arrows) compared among control, CUS, and FMC + CUS treated groups. It was noted that the spermatogonial cells are located on the seminiferous membrane.

It was shown that the TUNEL-positive cells were detected in the spermatogonial cells and were significantly increased in the CUS group compared to the control group. This apoptosis was dramatically decreased in the cotreatments with FMC extracts (40 and 80 mg/kg) compared to the CUS group, suggesting an anti-apoptotic effect of FMC in both doses (Figure 5). The antioxidant and anti-apoptotic effects of FMC extract may relieve testicular damage and improve poor sexual performance in rats under CUS conditions by decreasing lipid peroxidation, enhancing testosterone synthesis, protecting the testicular structure, and maintaining testicular function.

## 4. Discussion

This recent study has demonstrated the protective effect of FMC extract, which contains antioxidant capacity, on sexual dysfunction and testicular damage in chronic stress (CS) rats with the condition. Indeed, the FMC extract used in our study contained not only phenolic compounds and flavonoid substances but also various metabolites, including isoleucine, gamma-aminobutyric acid, choline, valine, alanine, and pyrimidine, which are essential for spermatogenesis, sperm motility, and acrosome reaction as previously described [34,35,36,37,38]. The sexual behavior parameters of male rodents have been used to test for the sexual motivation or libido capability [24,29,39,40]. This study demonstrated the improvements in sexual performance in CS rats treated with FMC extracts (Figure 2). It could be explained that FMC might protect the damage of parts of the brain responsible for sexual desires and enhance testosterone production, as shown in our results (Table 3). The increased testosterone levels have been revealed to associate with enhancing sexual behaviors in stressed animals treated with plant extracts containing bioactive compounds [6,39,40]. Additionally, a previous study demonstrated that MC fruit extract could stimulate testosterone synthesis in rats [41]. Moreover, the increased expressions of testicular CYP11A1 and StAR proteins in CS treated with FMC in our study confirmed the increase in serum testosterone levels (Figure 4). It was assumed that the antioxidant and bioactive compounds found in FMC could also protect Leydig cells from damage caused by CS, as low-dose FMC significantly decreased the MDA level in the CS testis (Table 3).

Previous studies have shown that CS can decrease carbohydrate and lipid metabolism, including the modulation of food intake-related hormones such as leptin, ghrelin, and insulin [42,43]. Interestingly, the aqueous extract of MC has been shown to increase the insulin levels in mice [44]. In this study, we therefore assumed that the improvement of final body weight in FMC-treated groups might result from the increased insulin level (Table 3). It was reported in previous studies [45,46,47,48,49] that CS can significantly increase the serum cortisol levels. Intriguingly, many studies, including this recent one, have shown no difference in cortisol levels between non-CS and CS rats [50,51,52,53]. Possibly, it was explained that CS may impair the glucocorticoid negative feedback, leading to HPA-axis hypoactivity [50]. Moreover, the fluctuation of cortisol level determinations might be caused by different intervals of time of the day during blood collection [52]. The decrease in testicular weights of the CS group in our study was agreed with previous works [6,12,54]. It was suggested that the reduction in epithelial height and tubular diameter in the CS condition was associated with the weight loss [54]. The FMC could prevent weight loss and improve testicular weight as well as morphometrics in CS rats (Table 3). Such improvements may corroborate the increase in DSP and decrease in testicular apoptosis as demonstrated in our study (Figure 3 and Figure 5). Previous studies have revealed that the CS increased the apoptosis in seminiferous tubules and decreased sperm concentration [13,15]. Our recent study showed the potential effects of the FMC on the decrease in apoptotic cells, MDA level, and cleaved caspase-3 expression in CS testes (Table 3; Figure 4 and Figure 5). Those protective effects of FMC against CS induced may be the result of its antioxidant capacities, as shown in Table 1. The CS was reported to activate apoptosis in the testis via both intrinsic and extrinsic pathways [13], where the expression of cleaved caspase-3 and -9 is increased in this pathway. In this study, the cleaved caspase-3 expression was increased, whereas that of caspase-9 was decreased in CS groups. It is known that caspases 3 and 9 are involved in intrinsic apoptosis, whereas cleaved caspases 3 and 8 are increased in the extrinsic pathway. Therefore, the decrease in cleaved caspase-9 in the CUS testis is still unexplained. However, both increased MDA level and cleaved caspase-3 expression in testicular tissues were initially used to confirm the apoptosis status (Table 3; Figure 4). It was then assumed that the extrinsic apoptosis pathway was involved in the CUS testis. To clarify this issue, the activation of caspase-8 and others must be further investigated.

Under chronic stress conditions, the hypothalamus–pituitary–adrenal (HPA) axis is activated, leading to the elevation of cortisol levels. The high cortisol level can increase free radicals, such as reactive oxygen species (ROS), as well as cause cellular oxidative stress, which leads to apoptosis in male germ cells by activating the apoptosis pathway [8,13,14,15]. In addition, the high cortisol level also decreased the testosterone level and reduced the sexual performance [55]. This study revealed the antioxidant activity and anti-apoptotic effects of FMC, which may reduce ROS levels and apoptosis in the CUS testis (Figure 4 and Figure 5). Moreover, the FMC could protect the Leydig cells from oxidative stress as well as significantly increase the testosterone level, which could improve sexual behaviors in the CUS group (Figure 2).

This study revealed that the FMC extract contains bioactive compounds such as choline that improve sperm concentration (Table 2; Figure 1). FMC also significantly improved the sexual parameters, including mount and intromission frequency, in stressed rats (Figure 2). In addition, FMC could protect against weight loss, significantly increase the serum testosterone level, and sperm production (Table 3; Figure 3). Moreover, the FMC could increase testicular morphometric parameters, including tubular diameter and epithelial height, as well as decrease the MDA level, TUNEL positive cells, and cleaved caspase 3 expression in stress groups, implying the protective effects of FMC on the testicular damage in chronic stress conditions (Table 3; Figure 4 and Figure 5).

Many previous studies showed that chronic stress has adverse effects on the male reproductive system and leads to male infertility [5,6,7,8,9,10]. Some studies have shown that *Momordica charantia* has anti-chronic stress effects in both human and animal models; however, none of these studies have revealed its effects on male fertility [56,57]. This study revealed that the FMC extract could reduce testicular damage and sexual dysfunction caused by chronic stress conditions and showed its potential for use as an alternative medicine for treating male infertility in future clinical studies.

## 5. Conclusions

This study demonstrated that FMC extract, rich in antioxidants and various bioactive compounds, could prevent testicular damage in CUS rats by reducing oxidative stress and inhibiting apoptosis. It also increased testosterone production and sexual performance in CUS rats. The FMC is a possible substance to be promoted as a supplement or alternative drug for enhancing male reproductive parameters, particularly in patients with chronic stress, anxiety, and depressive disorders. However, the optimal dose and the side effects of prolonged FMC use for more than 56 days should be evaluated in further studies.

## 6. Future Research Directions

This study revealed the preventive effects of FMC on sexual dysfunction and testicular damage in CUS rats. However, to advance FMC to clinical trials, studies are required to determine the optimal dose of FMC and assess its safety when used as an anti-infertility medicine over prolonged periods. Furthermore, the studies are needed for in-depth specific mechanisms as well as to identify the exact FMC bioactive compound response for its protective effect on sexual dysfunction.

## Figures and Tables

**Table 1:** The total phenolic content (TPC), flavonoid content (FC), and antioxidant activities using DPPH or FRAP assays of FMC extract compared to standard antioxidant agents. Data reported as mean ± SD.

Sample	Total Phenolic Content (mg GAE/g Sample)	Flavonoid Content (mg Catechin/g Sample)	DPPH Assay (mg Trolox Equivalents/g Sample)	DPPH Assay (IC50 mg/mL)	FRAP Value (µmol of Fe (II)/g Sample)
FMC	19.0 ± 0.27	0.31 ± 0.01	4.99 ± 0.09	2.01 ± 0.01	23.70 ± 0.82
Ascorbic acid	-	-	1554.62 ± 20.71	0.01 ± 0.00	12,390.55 ± 33.91
α-Tocopherol	-	-	667.12 ± 6.38	0.02 ± 0.01	4037.75 ± 80.75
BHT	-	-	183.05 ± 2.09	0.12 ± 0.00	2575.05 ± 41.42

GAE = gallic acid equivalent; DPPH = 2,2-diphenyl-1-picrylhydrazyl; FRAP = ferric reducing antioxidant power; IC50 = Half maximal inhibitory concentration of the DPPH radicals; FMC = fruit extract of *Momordica charantia*; BHT = Butylated hydroxytoluene.

**Table 2:** Lists of all metabolites identified in FMC extract by ^1^H NMR spectroscope with their chemical shift, multiplicity, STOCSY, and their *p*-values.

NO.	Chemical Shift (ppm)	Multiplicity	STOCSY	*p*-Value	Metabolites
1	0.93281	t	0.93281 (t), 1.01(d), 1.259 (m), 1.4562 (m), 1.985 (m), 3.662 (d)	1 × 10^−12^	Isoleucine
2	0.97536	t	0.97536 (t), 1.887 (m), 3.705 (dd)	1 × 10^−14^	2-aminobutyric acid
3	1.0425	d	0.980 (d), 1.042 (d), 2.233 (m), 3.59 (d)	1 × 10^−13^	Valine
4	1.1427	d	1.1427(d), 3.415 (dd), 3.546 (dd), 3.791 (m)	1 × 10^−13^	Propylene glycol
5	1.1666	m	1.1666 (m), 1.3199 (m), 1.703 (m), 3.873 (d)	1 × 10^−13^	Hydroxy-3-methylvaleric acid
6	1.203	s	1.203 (s)	1 × 10^−11^	Methylmalonic acid
7	1.4745	d	1.4745 (d), 3.759 (q)	1 × 10^−13^	Alanine
8	1.6995	m	0.94 (t), 1.6995 (m), 4.026 (dd)	1 × 10^−13^	Alpha-hydroxy
9	1.8903	m	1.8903 (m), 2.274 (t), 2.973 (t)	1 × 10^−13^	Gamma-aminobutyric acid
10	2.1012	s	2.1012 (s)	1 × 10^−10^	Acetic acid
11	2.1793	m	1.338 (m), 1.52 (m), 1.61 (m), 2.1793 (m), 2.509 (m)	1 × 10^−12^	Saccharopine
12	2.3862	dd	2.3862 (dd), 2.6 (dd), 4.292 (dd)	1 × 10^−12^	Malic acid
13	3.2155	s	3.2155 (s), 3.517 (m), 4.107 (m), 3.2713 (dd), 3.415 (m), 3.474 (m), 3.565 (dd)	1 × 10^−13^	Choline
14	3.2713	dd	3.72 (m), 3.889 (m), 4.026 (dd), 4.646 (d), 5.275 (d)	1 × 10^−13^	Glucose
15	3.6165	s	3.617 (s)	1 × 10^−14^	Propanedinitrile
16	3.632	s	3.632 (s)	1 × 10^−14^	Oxalacetic acid
17	4.102	m	3.824 (s), 3.961 (t), 4.102 (m), 4.55 (d)	1 × 10^−13^	6-phosphogluconic acid
18	6.531	s	6.531 (s)	1 × 10^−14^	Fumarate
19	8.481	s	8.481 (s)	1 × 10^−14^	Formic acid
20	9.142	d	9.1418 (s), 8.848 (d), 7.595 (td)	1 × 10^−11^	Pyrimidine
21	5.8222	d	7.88 (s), 5.8222 (d), 4.378 (dd), 4.255 (q), 3.872 (m), 3.791 (m)	1 × 10^−11^	Xanthosine
22	7.5948	d	8.2657 (m), 7.595 (d), 6.933 (d), 3.902 (d)	1 × 10^−11^	4-Aminohippuric acid
23	7.4317	m	7.389 (d), 7.4137 (m)	1 × 10^−11^	Cinnamic acid
24	8.0389	d	8.039 (d), 8.089 (d), 8.118 (d)	1 × 10^−11^	Adenine

S = single, d = doublet, t = triplet, m = multiplet, dd = double of doublet, q = quartet.

**Table 3:** Comparisons of body and testicular weights, sperm quality, serum hormone levels, testicular MDA levels, and testicular morphometrics among groups.

	Control	CUS	FMC40 + CUS	FMC80 + CUS
Body weight				
Initial BW (g)	381.33 ± 8.43	382.43 ± 12.71	377.71 ± 9.36	382.83 ± 18.10
Final BW (g)	478.22 ± 23.66	416.32 ± 22.97 ****	425.13 ± 6.58	460.87 ± 10.74 ^##^
Percentage change in BW	25.42 ± 5.83	10.62 ± 3.06 **	14.25 ± 5.37	19.54 ± 4.07
Testicular weight				
Absolute weight (g)	1.887 ± 0.10	1.693 ± 0.14 ***	1.841 ± 0.09 ^#^	1.881 ± 0.15 ^###^
Relative weight (g/100 g)	0.407 ± 0.01	0.397 ± 0.00	0.434 ± 0.01 ^###^	0.433 ± 0.02 ^###^
Sperm quality				
Sperm count (×10^6^)	28.790 ± 1.18	24.229 ± 4.80	40.700 ± 4.19 ^###^	38.012 ± 5.88 ^#^
Sperm viability (%)	95.667 ± 3.69	93.333 ± 1.04	94.167 ± 1.61	91.000 ± 4.77
Serum hormones				
Cortisol level (ng/mL)	1086.670 ± 248.46	1170.000 ± 52.92	993.330 ± 151.44	1436.670 ± 49.33
Testosterone level (ng/mL)	1.930 ± 0.04	0.679 ± 0.12 ****	1.078 ± 0.18 ^##^	1.163 ± 0.01 ^###^
Morphometrics				
Tubular diameters (μm)	295.949 ± 53.23	266.346 ± 37.77 *	302.169 ± 50.85 ^##^	300.336 ± 54.70 ^#^
Epithelial heights (μm)	66.410 ± 11.75	57.406 ± 11.61 ***	71.308 ± 15.01 ^####^	70.395 ± 17.29 ^####^
Testicular MDA level (ng/mg.protein)	0.663 ± 0.17	1.047 ± 0.14 *	0.511 ± 0.11 ^##^	0.987 ± 0.17

* *p* < 0.05, ** *p* < 0.01, *** *p* < 0.001, **** *p* < 0.0001 compared to control group, ^#^ *p* < 0.05, ^##^ *p* < 0.01, ^###^ *p* < 0.001, ^####^ *p* < 0.0001 compared to CUS.

## Data Availability

The data are not publicly accessible due to an ongoing study. However, the data used in this study can be obtained upon request from the corresponding author.
